# FSH-producing pituitary neuroendocrine tumor as a cause of ovarian hyperstimulation syndrome

**DOI:** 10.1530/EDM-23-0119

**Published:** 2024-02-28

**Authors:** Takuya Kitamura, Kazutaka Nanba, Kento Doi, Naoya Kishimoto, Kaoru Abiko, Ryo Kuwahara, Koki Moriyoshi, Naoko Inoshita, Tetsuya Tagami

**Affiliations:** 1 Department of Endocrinology and Metabolism, National Hospital Organization Kyoto Medical Center, Kyoto, Japan; 2 Department of Endocrinology, Metabolism, and Hypertension Research, Clinical Research Institute, National Hospital Organization Kyoto Medical Center, Kyoto, Japan; 3 Department of Neurosurgery, National Hospital Organization Kyoto Medical Center, Kyoto, Japan; 4 Department of Obstetrics and Gynecology, National Hospital Organization Kyoto Medical Center, Kyoto, Japan; 5 Department of Radiology, National Hospital Organization Kyoto Medical Center, Kyoto, Japan; 6 Department of Diagnostic Pathology, National Hospital Organization Kyoto Medical Center, Kyoto, Japan; 7 Department of Pathology, Moriyama Memorial Hospital, Tokyo, Japan

**Keywords:** Adult, Female, Asian - Japanese, Japan, Pituitary, Gynaecological endocrinology, Neuroendocrinology, Tumours and neoplasia, Insight into disease pathogenesis or mechanism of therapy, February, 2024

## Abstract

**Summary:**

Functioning gonadotroph tumors are rare neoplasms that can cause ovarian hyperstimulation syndrome (OHSS) in women of reproductive age. Here, we present a case of a follicle-stimulating hormone (FSH)-producing pituitary neuroendocrine tumor (PitNET) with irregular menstrual cycles and OHSS in a Japanese woman. A 34-year-old woman with bilateral multi-cystic ovarian mass was referred to our hospital for ovarian surgery. The imaging feature of magnetic resonance imaging (MRI) of the ovary and elevated estradiol levels with normal FSH and low luteinizing hormone (LH) levels led us to suspect the presence of a functioning gonadotroph PitNET. MRI revealed a 19-mm pituitary tumor, and increased tracer uptake was observed in the pituitary lesion on ^111^In-pentetreotide scintigraphy. Transsphenoidal tumor resection resulted in the resolution of the ovarian enlargement, normalization of her menstrual cycles, and spontaneous pregnancy. Immunohistochemistry (IHC) of the resected tumor for pituitary transcription factors, including steroidogenesis factor 1 (SF1) and estrogen receptor alpha, demonstrated positive immunoreactivity, whereas IHC for pituitary-specific positive transcription factor 1 was negative, suggesting that the tumor belonged to the SF1 lineage of PitNETs (gonadotroph tumor). The tumor cells showed positive expression of FSHβ, while LHβ was mostly negative. Consistent with the high pituitary tumor uptake observed on ^111^In-pentetreotide scintigraphy, the pituitary tumor showed positive expression of somatostatin receptor 2A. Detailed clinical and histological evaluations will provide useful information to understand these rare functioning gonadotroph tumors better.

**Learning points:**

- Functioning gonadotroph tumors are very rare neuroendocrine tumors of pituitary origin.
- Women of reproductive age presenting with bilateral multi-cystic ovarian enlargement, irregular menstrual cycles, and hyperestrogenemia under unsuppressed follicle-stimulating hormone (FSH) levels should be evaluated for FSH-producing tumor.
- Raising awareness of OHSS due to functioning gonadotroph tumors is crucial to prevent unnecessary ovarian surgery.
- Comprehensive histological analysis may provide useful information to better understand the characteristics of functioning gonadotroph tumors.

## Background

Pituitary neuroendocrine tumors (PitNETs), also known as pituitary adenomas, can be classified as functioning tumors that cause hormone excess syndrome or non-functioning tumors. Gonadotroph tumors account for approximately 75% of non-functioning PitNETs and 40% of clinically recognized macroadenomas (1). However, functioning gonadotroph tumors are very rare (2). The clinical manifestations of functioning gonadotroph PitNETs include menstrual disorders, infertility, ovarian hyperstimulation syndrome (OHSS) in premenopausal women and adolescent girls, testicular enlargement in men, and precocious puberty in children (3). OHSS, which usually occurs in women undergoing assisted reproductive techniques, is an exaggerated response to gonadotropin stimulation characterized by multi-cystic enlargement of the ovaries associated with pain and abdominal bloating (4). In premenopausal women with functioning gonadotroph tumors, ovarian enlargement can be resolved after transsphenoidal surgery, leading to the normalization of menstrual cycles and a successful pregnancy (3). Therefore, early detection is important to avoid unnecessary ovarian surgery. Herein, we present a case of a functioning gonadotroph PitNET with OHSS that was resolved after removing the pituitary tumor.

## Case presentation

**Figure 1:**
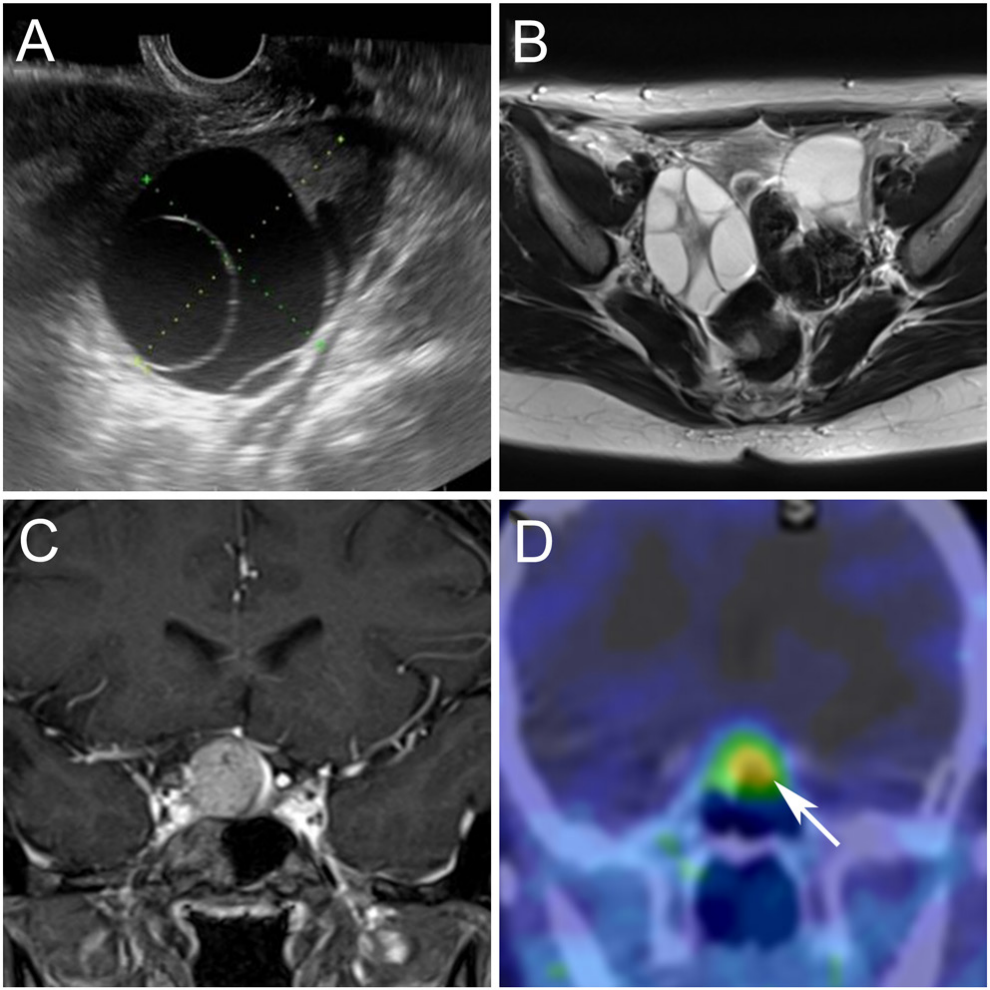
Preoperative imaging findings of ovary and pituitary lesions. (A) Transvaginal ultrasound showed a large septated cystic ovary. (B) Pelvic MRI (axial T2-weighted image) showed bilateral multi-cystic ovaries. (C) preoperative pituitary MRI (coronal postcontrast T1-weighted image) revealed a mass lesion with suprasellar extension. (D) Preoperative ^111^In-pentetreotide scintigraphy showed increased tracer uptake in the pituitary lesion (arrow).

A 34-year-old Japanese woman with a history of irregular menstrual cycles was referred to our hospital for surgical intervention for an ovarian mass. She experienced menarche at the age of 13, and her menstrual cycle was regular until the age of 27. The patient had no history of precocious puberty. Transvaginal ultrasonography revealed a multi-cystic ovarian mass of 6 and 7 cm diameters in the right and left ovaries, respectively (Fig. 1A). On magnetic resonance imaging (MRI), numerous enlarged intraovarian cysts with high signal intensity on T2-weighted images were observed (Fig. 1B). Based on MRI findings, OHSS rather than ovarian tumors was suspected, and then an endocrine work-up was performed.

## Investigation

The laboratory test results are summarized in Table 1. Elevated serum prolactin (44.3 ng/mL) and estradiol levels (494 pg/mL) were observed. The serum follicle-stimulating hormone (FSH) level was within the normal range (8.4 mIU/mL) despite elevated estrogen levels, whereas the serum luteinizing hormone (LH) level was low (0.7 mIU/mL). The serum human chorionic gonadotropin (hCG) level was <0.2 mIU/mL. Based on the endocrinological evaluation, OHSS due to gonadotropin excess was suspected. An MRI revealed a 19 mm pituitary tumor with moderate compression of the optic chiasm (Fig. 1C). Additionally, a visual field examination revealed the enlargement of Mariotte’s blind spots. An increased uptake in the pituitary lesion was observed on ^111^In-pentetreotide scintigraphy (Fig. 1D).

**Table 1:** Summary of pre- and postoperative hormone levels

	Reference range	Baseline before surgery		Postoperative follow-up data		
		11 weeks	2 weeks	4 weeks	8 weeks	15 weeks^**^
Free T3, pg/mL	1.68–3.67	NA	2.37	2.32	2.01	NA
Free T4, ng/dL	0.70–1.48	NA	0.80	0.96	0.87	NA
TSH, μU/mL	0.35–4.94	1.026	0.940	0.622	1.470	NA
PRL, ng/mL	4.91–29.32	44.3	29.8	6.0	9.1	12.6
GH, ng/mL	0.13–9.88	NA	0.52	0.99	2.69	1.03
IGF-1, ng/mL	115–277	NA	84	66	84	77
ACTH, pg/mL	7.2–63.3	NA	15.1	4.8	7.7	16.1
Cortisol, μg/dL	7.07–19.6	NA	7.1	4.9	5.9	6.7
DHEA-S, μg/dL	58–327	NA	122	119	94	114
LH, mIU/mL		0.7	0.4	2.2	6.8	3.2
Follicular	2.4–12.6					
Mid-cycle peak	14.0–95.6					
Luteal	1.0–11.4					
Postmenopausal	7.7–58.5					
FSH, mIU/mL		8.4	8.8	8.3	6.9	3.4
Follicular	3.5–12.5					
Mid-cycle peak	4.7–21.5					
Luteal	1.7–7.7					
Postmenopausal	25.8–134.8					
Estradiol, pg/mL		494	245	15	81	184
Follicular	19–226					
Mid-cycle peak	49–487					
Luteal	78–252					
Postmenopausal	≤39					
Progesterone, ng/mL		1.1	0.3	<0.1	0.1	11.8
Follicular	≤0.4					
Mid-cycle peak	≤3.7					
Luteal	8.5–21.9					
Total testosterone, ng/mL	0.06–0.86	0.19	0.19	0.15	0.22	NA
Inhibin B, pg/mL	35.6–139.1^*^	NA	281.4	12.1	NA	NA

^*^Reference interval was adopted from Wen *et al.* (14); ^**^Luteal phase.

ACTH, adrenocorticotropic hormone; DHEA-S, dehydroepiandrosterone sulfate; FSH, follicle-stimulating hormone; GH, growth hormone; IGF-1, insulin-like growth factor-1; LH, luteinizing hormone; NA, not available; PRL, prolactin; T3, triiodothyronine; T4, tetraiodothyronine; TSH, thyroid-stimulating hormone.

Based on the patient’s clinical presentation, laboratory test results, and imaging studies, she was diagnosed with an FSH-producing PitNET. Repeated laboratory tests revealed serum estradiol of 245 pg/mL with normal FSH (8.8 mIU/mL) and low LH (0.4 mIU/mL). The plasma inhibin B level was elevated (281.4 pg/mL). The GH level was normal, whereas the IGF-1 level was low (Table 1).

## Treatment

**Figure 2:**
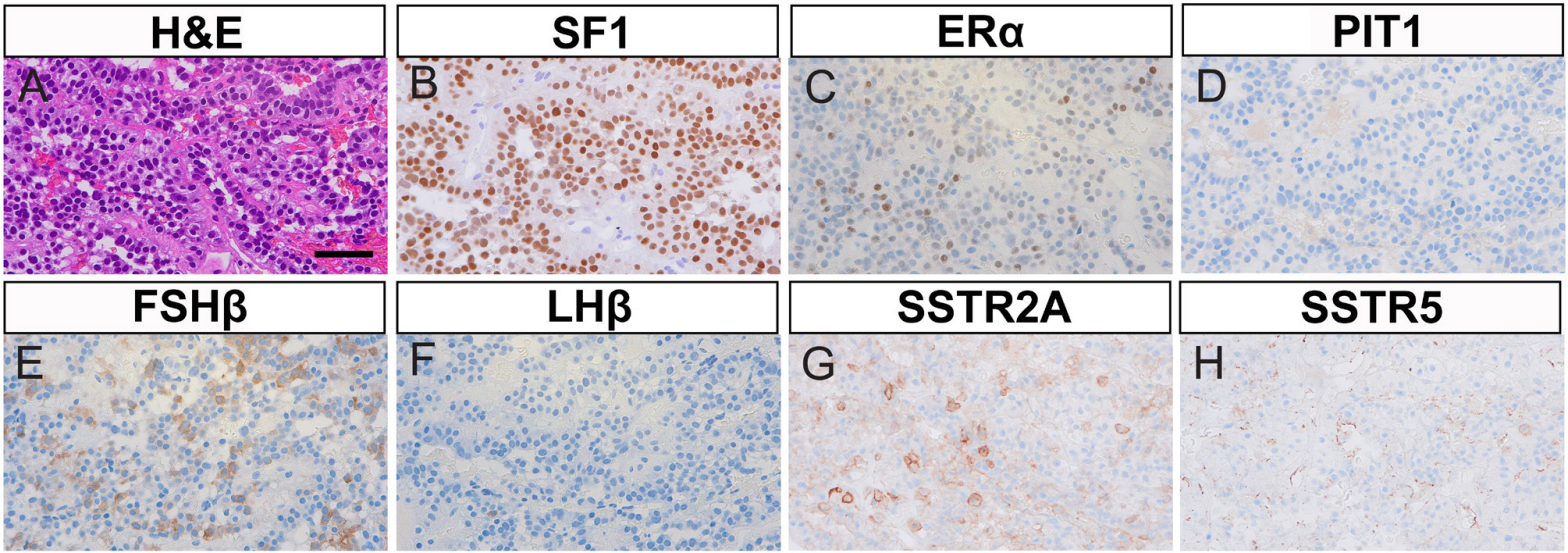
Histopathology of resected pituitary tumor. High-magnification images of the resected pituitary tumor are shown (40× objective lens). (A) Hematoxylin and eosin staining (H&E), scale bar 50 µm. (B–D) Immunohistochemistry (IHC) for pituitary transcription factors. (B) Steroidogenic factor 1 (SF1). (C) Estrogen receptor alpha (ERα). (D) Pituitary-specific positive transcription factor 1 (PIT1). (E and F) IHC for gonadotropins. (E) Follicle-stimulating hormone subunit beta (FSHβ). (F) Luteinizing hormone subunit beta (LHβ). (G and H) IHC for somatostatin receptor (SSTR) subtypes. (G) SSTR2A. (H) SSTR5.

The patient underwent endoscopic transsphenoidal surgery, and the soft yellowish tumor was resected. Hematoxylin and eosin staining revealed proliferating homogeneous cells with round nuclei that formed a trabecular structure and perivascular pseudorosette formations, indicative of a PitNET (Fig. 2A). The Ki-67 labeling index was 0.2%, and no obvious mitotic figures were observed. Immunohistochemistry (IHC) of the resected tumor for steroidogenesis factor 1 (SF1) and estrogen receptor alpha (ERα) demonstrated that tumor cells were positive in the nucleus (Fig. 2B and C), while pituitary-specific positive transcription factor 1 (PIT1) was negative (Fig. 2D), suggesting a gonadotroph tumor (SF1 lineage PitNET). As for hormonal staining, cytoplasmic FSHβ immunoreactivity was observed, while LHβ was mostly negative (Fig. 2E and F). Furthermore, somatostatin receptor 2A (SSTR2A) showed membrane expression, whereas no significant immunoreactivity for SSTR5 was observed (Fig. 2G and H).

## Outcome and follow-up

There were no major postoperative complications. Hydrocortisone was administered perioperatively to prevent adrenal insufficiency and was discontinued at discharge. One month after surgery, serum estradiol level decreased (15 pg/mL), and serum FSH and LH levels were 8.3 mIU/mL and 2.2 mIU/mL, respectively. Moreover, plasma inhibin B fell to 12.1 pg/mL (Table 1). Postoperatively, a visual field examination showed improvement in the enlargement of Mariotte’s blind spot. Two months after surgery, the ovarian size reduced to 2.7 cm in diameter. The patient’s menstrual cycles resumed 12 weeks after surgery. Half a year after the surgery, she achieved spontaneous pregnancy.

## Discussion

Here, we present a rare case of a functioning gonadotroph PitNET that led to OHSS. In this case, ovarian surgery was initially planned because of suspicion of ovarian tumors but was ultimately avoided after careful evaluation. In women of reproductive age with bilateral multi-cystic ovarian enlargement, the concurrent presence of elevated estradiol levels with unsuppressed FSH and low LH should prompt brain imaging to find a functioning gonadotroph PitNET. When premenopausal patients with functioning gonadotroph tumors are classified into FSH- or LH-predominant types according to their preoperative serum FSH/LH ratios, 92.3% and 7.7% are FSH-predominant (FSH/LH ratio >1) and LH-predominant (FSH/LH ratio <1), respectively (5). Of the FSH-predominant types of functioning gonadotroph tumors, menstrual disorders, infertility, and OHSS were reported in 86.7%, 16.7%, and 98.2% of cases, respectively (5).

So far, more than 70 cases of functioning FSH-producing PitNETs in premenopausal women have been reported in the literature (5, 6, 7, 8, 9). The average age of patients diagnosed with functioning FSH-producing PitNETs is in the early 30s (5). There is typically a delay of approximately 3 years in diagnosis, and approximately half of the patients experience ovarian surgery before the diagnosis (5). Functioning FSH-producing PitNETs can be found in postmenopausal women as well as adult men and children. In postmenopausal women, the clinical findings are similar to those of a non-functioning PitNET because the ovaries do not respond to FSH stimulation. Since the increase in gonadotropins is secondary to menopause, low LH levels or discrepancy between FSH and LH could be indicative of a functioning FSH-producing PitNET in postmenopausal women but not specifically (3). In men, functioning FSH-producing PitNET can cause testicular enlargement due to stimulation of the seminiferous tubules by FSH (3). In children with functioning FSH-producing PitNET, isosexual precocious puberty has been reported in both girls and boys diagnosed between the ages of 3 and 7 years (3). Ovarian enlargement can be present in girls, and enlargement of the testes can also occur in affected boys (3).

Notably, a normal FSH level does not always exclude the possibility of functioning gonadotroph tumors. Instead, suppressed LH and high estradiol levels can be considered the characteristic endocrinological profile of FSH-secreting pituitary tumors (5). In the present case, the 11-week and 2-week preoperative data showed fluctuating levels of estradiol and prolactin in the conditions of normal FSH and suppressed LH levels (Table 1). A similar case of FSH-producing pituitary tumor showing fluctuated estradiol levels has been reported in the literature (10). The fluctuation in estradiol may partly be explained by irregular follicle recruitment and atresia. Moreover, prolactin is regulated facilitatively by estradiol (11). Therefore, these may explain the fluctuations in estradiol and prolactin in this case.

Several hypotheses have been proposed to explain why relatively low FSH levels can cause florid clinical manifestations, such as OHSS. First, slight but constant FSH release and alteration of the pulsatile secretion of gonadotropins could stimulate the recruitment of multiple dominant follicles and the release of high serum estradiol, similar to that of OHSS induced by exogenous FSH administered for fertility treatment (3). Therefore, in patients with FSH-producing pituitary tumors, increased serum estradiol concentrations can suppress the hypothalamus–anterior pituitary gland axis by a negative feedback mechanism, reducing excessive FSH production to normal levels. Moreover, the serum LH concentration could be reduced below the lower limit of the normal range by the negative feedback mechanism or by compression of the normal pituitary gland by the tumor (3, 12). Another hypothesis is that alterations in the alpha and beta chains of the heterodimer could potentially lead to increased bioactivity of FSH (13). In the present case, plasma inhibin B level was elevated preoperatively (281.4 pg/mL), and a marked decrease was observed postoperatively (12.1 pg/mL). Inhibins are a class of proteins produced by the ovary in women, and their primary functions include the regulation of pituitary FSH secretion and ovarian steroidogenesis via autocrine/paracrine mechanisms. Specifically, inhibin B is mainly produced during the follicular phase of the menstrual cycle and inhibits FSH secretion during this phase (14). Like our case, elevated inhibin B levels have been observed in patients with FSH-producing tumors presumably in association with FSH stimulation (15).

According to the World Health Organization 2022 classification, gonadotroph tumors belong to the SF1 lineage and are immunoreactive for FSH, LH β-subunits, alpha subunit (α-SU), SF1, ERα, and GATA-3 (2). In the present case, the results of IHC for pituitary transcription factors and gonadotropins were compatible with FSH-producing PitNETs. Consistent with a previous report by Ichijo *et al.* (12), the tumor exhibited high SSTR2A and low SSTR5 expression. ^111^In-pentetreotide is a radiolabeled somatostatin analog used in somatostatin receptor scintigraphy. The increased uptake of ^111^In-pentetreotide in functioning gonadotroph tumors has been previously documented (4). Notably, ^111^In-labeled pentetreotide binds specifically to SSTRs with a particular affinity for subtypes 2 and 5 (16). Therefore, ^111^In-pentetreotide scintigraphy before surgery may be useful for evaluating SSTR expression, which is generally associated with the therapeutic response to somatostatin receptor ligands (SRLs) (16). In our case, increased tumor uptake was documented on ^111^In-pentetreotide scintigraphy, consistent with the positive immunoreactivity of SSTR2A in the resected tumor.

Owing to the rarity of the disease, there are no existing guidelines providing recommendations regarding the optimal management of functioning FSH-producing PitNETs (3). Transsphenoidal surgery is the primary treatment for the hormone excess syndrome and its pressure effects. Adjuvant radiotherapy can be offered in selected cases, such as those with postoperative residual tissue (3). Medical therapies such as SRL and dopamine agonists appear to be effective only in limited cases, and there are no definitive markers to predict their therapeutic effects (3). Thus, dedicated clinical studies are needed to determine the factors that predict the efficacy of SRL and dopamine agonists on functioning gonadotroph tumors.

In conclusion, we present a rare case of an FSH-producing PitNET that caused OHSS. In such cases, an accurate diagnosis is the key to avoiding unnecessary ovarian surgeries. A comprehensive histological analysis will provide useful information for better characterization of functioning gonadotroph tumors.

## Declaration of interest

KN received a research grant from AstraZeneca, which is unrelated to the current work. All authors declare that there is no conflict of interest that could be perceived as prejudicing the impartiality of the case study reported.

## Funding

This work was partially supported by grants from the Japan Society for the Promotion of Sciencehttp://dx.doi.org/10.13039/501100001691 KAKENHI (grants no. JP18K11093 and JP23K07980 to TT) and the Takeda Science Foundationhttp://dx.doi.org/10.13039/100007449 (to K.N).

## Patient consent

Written informed consent for publication of their clinical details and clinical images was obtained from the patient.

## Author contribution statement

All authors contributed individually to authorship. TK and KN drafted the manuscript. TK, KD, and NK were directly involved in the patient care. RK performed the radiological assessment. KM and NI performed histological analysis. KN, KA, and TT provided input for the patient care and case report. All authors reviewed and approved the final draft of the manuscript.
